# Metabolomic Profiling of Soybeans (*Glycine max* L.) Reveals the Importance of Sugar and Nitrogen Metabolism under Drought and Heat Stress

**DOI:** 10.3390/plants6020021

**Published:** 2017-05-25

**Authors:** Aayudh Das, Paul J. Rushton, Jai S. Rohila

**Affiliations:** 1 Department of Biology and Microbiology, South Dakota State University, Brookings, SD 57007, USA; aayudhdas@gmail.com (A.D.); wrkypaul1@gmail.com (P.J.R.); 2 Department of Plant Biology, University of Vermont, Burlington, VT 05405, USA; 3 22nd Century Group Inc., Clarence, NY 14031, USA; 4 Dale Bumpers National Rice Research Center, USDA-ARS, Stuttgart, AR 72160, USA

**Keywords:** crop yield, drought stress, heat stress, metabolomic profiling, phytochemicals, soybean

## Abstract

Soybean is an important crop that is continually threatened by abiotic stresses, especially drought and heat stress. At molecular levels, reduced yields due to drought and heat stress can be seen as a result of alterations in metabolic homeostasis of vegetative tissues. At present an incomplete understanding of abiotic stress-associated metabolism and identification of associated metabolites remains a major gap in soybean stress research. A study with a goal to profile leaf metabolites under control conditions (28/24 °C), drought [28/24 °C, 10% volumetric water content (VWC)], and heat stress (43/35 °C) was conducted in a controlled environment. Analyses of non-targeted metabolomic data showed that in response to drought and heat stress, key metabolites (carbohydrates, amino acids, lipids, cofactors, nucleotides, peptides and secondary metabolites) were differentially accumulated in soybean leaves. The metabolites for various cellular processes, such as glycolysis, the tricarboxylic acid (TCA) cycle, the pentose phosphate pathway, and starch biosynthesis, that regulate carbohydrate metabolism, amino acid metabolism, peptide metabolism, and purine and pyrimidine biosynthesis, were found to be affected by drought as well as heat stress. Computationally based regulatory networks predicted additional compounds that address the possibility of other metabolites and metabolic pathways that could also be important for soybean under drought and heat stress conditions. Metabolomic profiling demonstrated that in soybeans, keeping up with sugar and nitrogen metabolism is of prime significance, along with phytochemical metabolism under drought and heat stress conditions.

## 1. Introduction

Environmental stresses, such as drought and heat, are serious threats to soybean cultivation in the US and in other soybean growing countries, causing multi-billion dollar losses every year. Stress in plants can arise as a reduction in growth and development that could be the result of disrupted metabolic homeostasis which requires the temporal readjustment of several metabolic pathways [1]. Under abiotic stress it is a general trend that growth and yield of crops are reduced below optimum levels [2]. Deryng et al. (2014) showed that by the year 2080, soybean yields at global level may reduce by a quarter (*ΔY* = 15.3 ± 26.5% versus 20.4 ± 22.1% without heat stress) as a result of heat stress [3]. The growth, development and yields of soybean are significantly affected by drought and heat stress; thus, in mid-south United States, an alternative production method, i.e., the Early Soybean Production System, is in practice to help farmers to shift the planting and harvest times by incorporating maturity group III–V in soybean cultivation to minimize late season drought stress effects on yield [4]. Further, it has also been shown that abiotic stresses are directly related to changes in various cellular metabolic pathways [4,5,6,7,8], including carbohydrate, amino acid, and peptide metabolism [9]. Hence, abiotic stresses that alter the metabolomic profiles of soybeans leaf tissues (source) could reduce its yield (sink) potential. In the past, genetic and agronomic advancements have enhanced the drought tolerance of crop plants, and have resulted in stabilizing yields over time [10], but now with advancements in mass spectrometry (MS) technology, it is imperative to study the global metabolomic responses of soybeans to drought and heat stresses for developing metabolomic markers, utilizing metabolic pathways, and assisting soybean breeding programs. Non-targeted metabolomics aided by the advancements of MS technologies is capable of profiling diverse classes of metabolites, and thus has become a platform of choice to study metabolites perturbed by environmental cues [5], and are continually adding significant understanding to our knowledge of molecular mechanisms that could lead to predictive biology in the future by taking a systems biology approach [6]. Recently, Rabara et al. [7] compared two metabolomic profiles of soybean and tobacco to elucidate unique signature metabolites during progressive dehydration treatment, and showed that 4-hydroxy-2-oxoglutaric acid in tobacco roots and coumestrol in soybean roots show the highest tissue-specific accumulations. Previously, in a similar progressive drought stress study in *Medicago truncatula*, Zhang et al. [11] meticulously combined transcriptomic and metabolomic data of roots and shoots from control, mild-, moderate-, severely-stressed, and re-watered plants, and provided insights into the regulation of metabolic pathways operating under different levels of drought stress in plants. The study concluded that myo-inositol and proline had striking regulatory profiles indicating involvement in the *Medicago* drought tolerance mechanism. 

Previously, our group reported differential proteome expression of soybean leaves under drought and heat stress conditions [12]. The present study was a logical extension of the proteomic evaluations, to the metabolomic level. Genetic variability within a crop species is strongly expressed at the metabolome level. In other words, the genotypic effect on the metabolome could be much greater than the analytical noise or within-group biological variations. Likewise, the magnitude of metabolic or proteomic variations for a single variety under constant conditions is typically so low that it can be used to easily detect many types of stress responses including drought and heat. 

Metabolomics is being used to measure differential abundance of metabolites at a global level; and has successfully been used in many crops such as soybean, rice, corn, and barley [13,14,15,16,17]. In Arabidopsis, metabolic profiling has elucidated the temporal dynamics associated with heat stress [18]. Earlier integrated metabolomic-proteomics studies have shown comprehensive exploration of soybean roots and hypocotyls under abiotic [19] and wheat biotic stresses [20]. Similar metabolomic studies were performed in grapes for drought and salinity stresses to define alterations in the metabolic pathways related to gluconeogenesis, energy metabolism and nitrogen assimilation [21]. At present an incomplete understanding of abiotic stress-associated metabolism and identification of associated metabolites remains a major gap in soybean stress research. In addition, metabolomic studies of economically important crops, such as soybean that is being used for food as well as feed, are of special interest, and are advantageous for genomics-assisted selection for plant breeders to develop improved germplasm and release novel climate resilient varieties in the future [22].

A previous report of metabolomic profiling of soybean leaves has been performed using gas chromatography-mass spectrometry (GC/MS) analysis [23]. GC/MS is a very powerful when focusing on primary metabolites whereas liquid chromatography-mass spectrometry (LC/MS) is suitable for the detection of secondary metabolites [17]. A combination of GC/MS- and LC/MS-based metabolite profiling study in *Medicago truncatula* showed a significant alteration in amino acid and carbohydrate metabolism in response to biotic and abiotic elicitors [24]. In our soybean metabolomic study of leaves using GC- and LC-coupled with MS, we determined differential abundances of various primary and secondary metabolites, such as carbohydrates, amino acids, lipids, cofactors, nucleotides, peptides, and secondary metabolites, in response to drought and heat stresses. We demonstrated that in response to drought and heat stress, key metabolites were differentially regulated in various metabolic processes that included: (1) glycolysis, the TCA cycle, the pentose phosphate pathway, and starch biosynthesis that regulates carbohydrate metabolism; (2) amino acid metabolism; (3) peptide metabolism; (4) purine and pyrimidine biosynthesis that regulates nucleotide metabolism as a whole; and (5) metabolism involving secondary metabolites, including essential phytochemicals. The results clearly reveal various metabolic regulations and changes in metabolic pathways that correlate with plant growth and development.

## 2. Results and Discussion

### 2.1. Differential Expression of Metabolites in Response to Heat and Drought Stress

**Figure 1:**
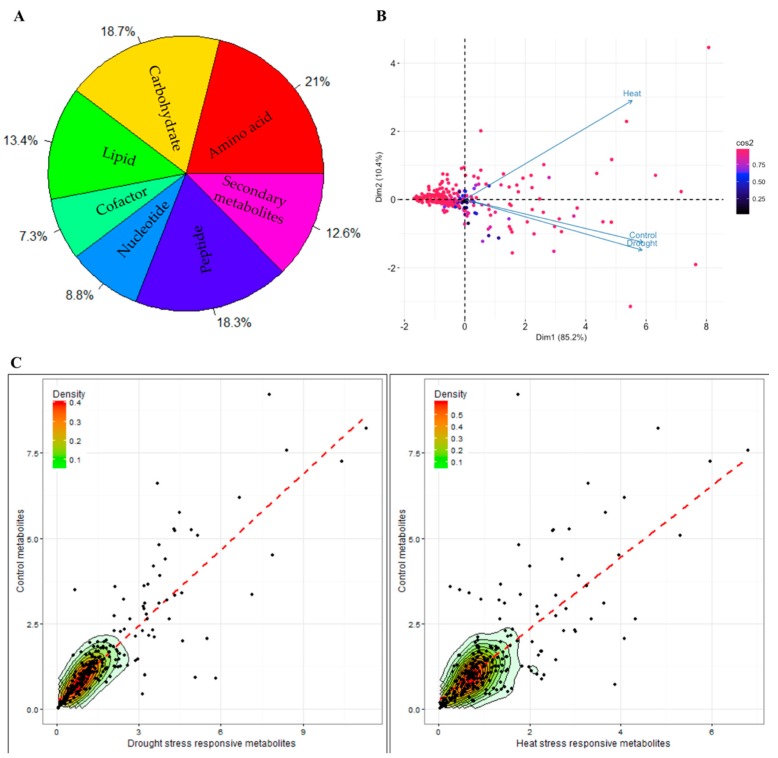
Metabolomics data of soybean leaves under drought and heat stress. (**A**) Classification of differentially expressed metabolites into chemical groups, in response to both drought and heat stress, based on their metabolic designations. Percentage values symbolize abundance of metabolites in each functional class. (**B**) Principal component analysis (PCA) biplot of all the variable metabolites originating from three different treatments i.e., control, drought and heat is shown here, (cos2 = the quality of the individuals on the factor map displayed as color intensity). (**C**) Scatter plot distribution of all groups of metabolites revealed that there is a linear positive correlation between the expression levels of control metabolites, and drought stress and heat stress-responsive metabolites. The best scatter of the metabolites was observed at the base of both the plot, which is visualized with an intensity level gradient ranging from 0.1 to 0.5. (Plot created by using R 3.3.1 software with the ggplot package).

Metabolites are the end products of the cellular regulatory processes, and their levels are regulated as biological system responses to environmental stress conditions. This analysis demonstrates that in response to drought and heat stresses the global metabolite present at a given time in plant cell “i.e., metabolome”, changes and show differential regulation when compared between the treatments. Figure 1A, Figure S1, and Table S1 show the differential abundance of metabolites detected by GC-MS and LC-MS. Our metabolomics profiling using GC-MS and LC-MS revealed the concurrent detection of 266 putative metabolites, including primary and secondary metabolites. Interestingly, this study demonstrated that under heat stress, the number of metabolites (carbohydrates, amino acids, lipids, cofactors, nucleotides, peptides and secondary metabolites) decreased to a greater extent than under drought stress.

A principal component analysis (PCA) was performed on metabolite data to identify a pattern between the samples. Figure 1B (biplot of variables) shows that metabolites have been clustered, as each vector represents the accumulated effect of all the metabolites in that particular treatment, differently in response to heat stress compared to the control samples. Drought stress was found to have less of an effect compared to heat stress, based on PCA analysis. A scatter plot analysis (Figure 1C) revealed that there was a linear positive relationship for metabolite expression among the drought and heat stresses when compared with the control condition. We fitted a linear model to ascertain whether it was statistically rational to fit a linear correlation to those variables and to compare their differential expression. Based on the statistical inference that was derived by comparing the control versus drought, and control versus heat stress metabolites, we determined that there was a positive linear relationship among the fold changes of metabolites between stress and the control conditions. 

### 2.2. Effect of Drought and Heat Stress on Carbohydrate Metabolism

**Figure 2:**
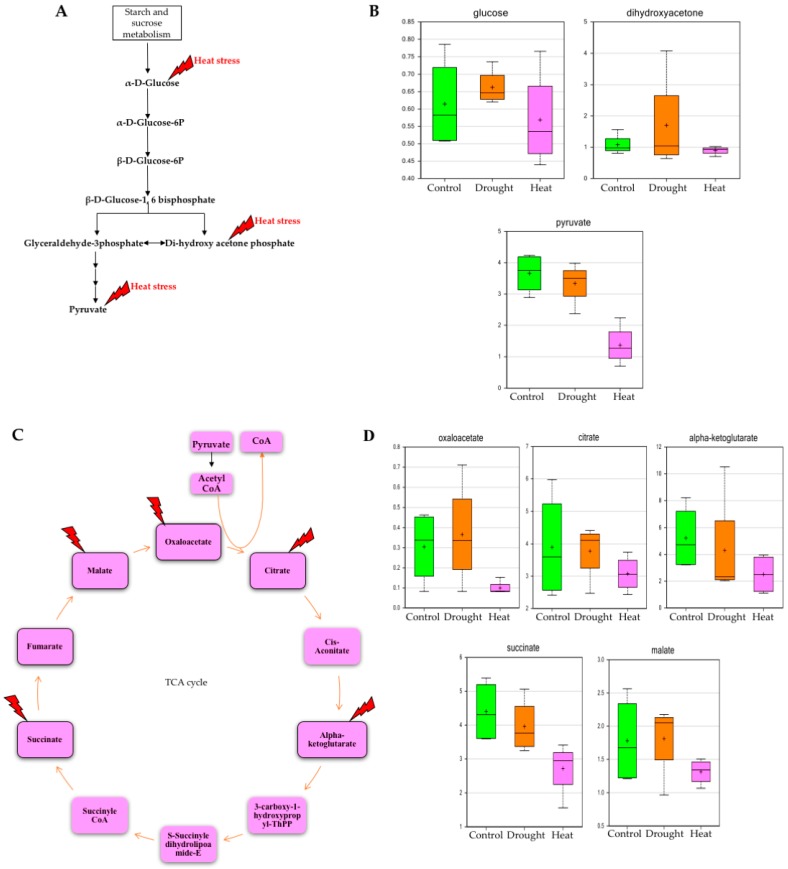
Effects of drought and heat stresses on the metabolites of glycolysis and the tricarboxylic acid (TCA) cycle. (**A**) Glycolysis pathway showing the metabolites that had abundances significantly affected by stresses in the study; (**B**) Box plots showing the relative abundances of glucose, dihydroxyacetone and pyruvate under the control, drought, and heat stress conditions. (**C**) TCA cycle showing metabolites that were found to have significant effect on their abundance of stress (**D**) Box plots showing relative abundances of metabolites of TCA cycle under various stressed conditions [the *Y* axis defines the relative abundances of specific metabolite and *X* axis defines the treatment group; the red lightning sign in panels (**A**,**C**) indicates heat stress; green box plot: control, orange box plot: drought and pink box plot: heat]. Inside the box plots, “+” indicates mean value, and “—“ indicates median value.

**Figure 3:**
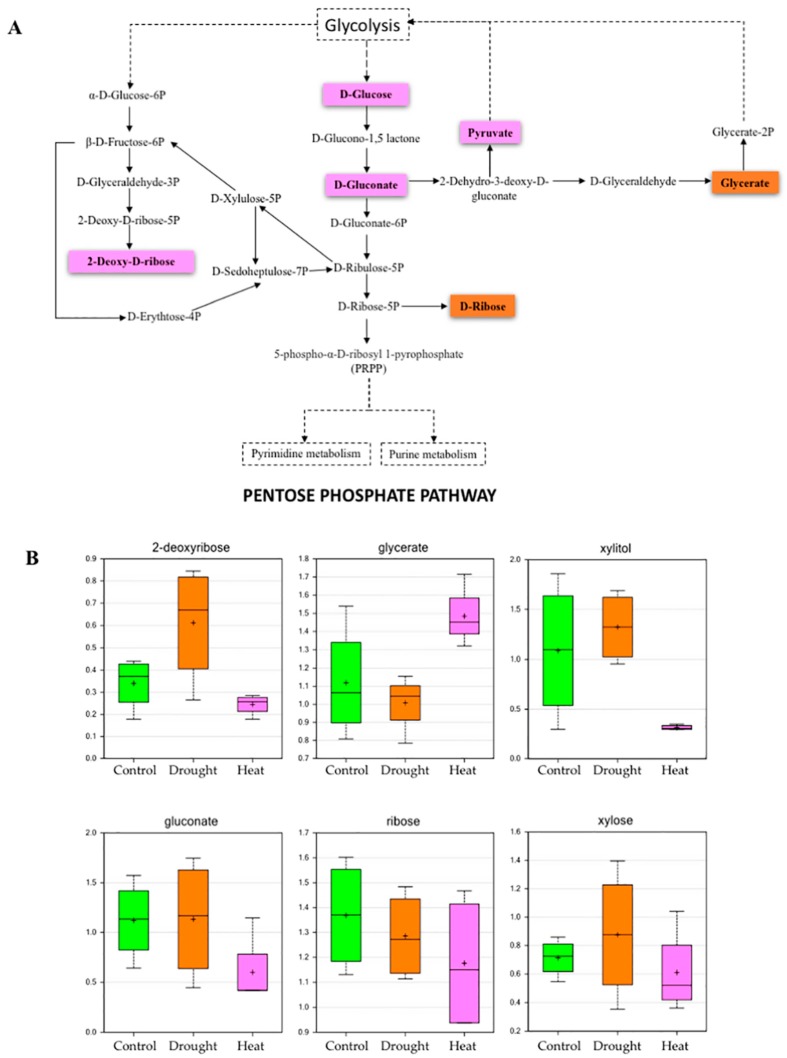
Effect of drought and heat stresses on metabolites related to the pentose phosphate pathway. (**A**) The pink colored metabolites indicate downregulation in response to heat stress, orange colored metabolites indicate downregulation in response to drought stress. (**B**) Box plots showing relative abundances of PPP metabolites and sugar compounds under control, drought, and heat stress conditions (pink highlights indicate heat stress-affected; box plot description is the same as Figure 2).

The rate of glucose utilization by cells is known to be influenced by various environmental factors [25]. One of the outcomes of the current study was to observe the effects of heat stress on carbohydrate metabolism, specifically in glycolysis and the pentose phosphate pathway (PPP). Oxidative PPP is a major source of reducing power and metabolic intermediates for several biosynthetic processes in the cell, and apparent change in the cellular abundance of certain PPP metabolites often severely affects several other metabolism pathways [26]. Analysis of this study revealed that during heat stress, the amount of major glycolysis and PPP metabolites, such as glucose, dihydroxy acetone phosphate and pyruvate, decreased drastically compared to the control treatment (Figure 2 and Figure 3, and Table S2). Respiratory pathways, such as glycolysis and the tricarboxylic acid (TCA) cycle, are essential for energy delivery to different organelles, and for maintaining various physiological functions [27]. The study indicates that during heat stress, the level of several TCA cycle metabolites, such as oxaloacetate, citrate, alpha-ketoglutarate, succinate and malate decrease significantly (Figure 2, and Table S2). This would be expected to cause a reduction in adenosine triphosphate (ATP), diminishing plant photosynthesis and carbon fixation, which may have correlating effects on soybean yields. Furthermore, proteomic studies [12] have also indicated that in response to drought and heat stresses several major enzymes of the Calvin cycle and PPP were highly affected. The metabolomic findings of the current study support such proteomic results, and illustrate how drought and heat stress may affect carbohydrate metabolism.

It has been reported that constant exposure of plants to high temperature stress reduces the consistent increase in starch and soluble sugar (e.g., maltose, sucrose) concentration, and decreases the soluble sugar concentrations in maturing pollen grains [28]. The results of this study also show that due to high air temperature (heat) stress, maltose, galacititol, sucrose, and mannitol concentrations decrease in leaves by more than two-fold (Figure S2). Primarily, due to a low sugar concentration, the transport of sugars to the reproductive cells may be greatly affected, resulting in poor development of the gametophyte [29]. 

The metabolic fate of maltose due to heat stress will ultimately be seen in glycolysis, as maltose is broken down into glucose followed by glucose-6-phosphate, which regulates glycolysis, 2-keto-3-deoxy-6-phosphogluconate (KDPG) and the pentose phosphate pathways [30]. Plants use mannitol, one of the crucial sugar alcohols that is synthesized simultaneously with sucrose, as an important photo-assimilate, and almost half the fixed CO_2_ is converted to mannitol, which is used during growth of non-photosynthetic [31] and sink tissues [32]. Interestingly, our results indicate that mannitol is reduced due to heat stress, which may affect phloem translocation in soybeans. Furthermore, galactitol, one of the major sugar alcohols produced by the reduction of galactose, was reduced during heat stress, which indicates a decrease in galactose that may directly affect glycolysis and other major metabolic pathways that use galactose. One study shows that under stress conditions, when galactose cannot be utilized routinely in a conventional way, galactitol is formed in tissues to prevent galactose toxicity [33]. In response to drought stress, we found that most of the major carbohydrates such as glucose, fructose, sucrose and raffinose were accumulated at a higher level as compared to the control, which indicates that in response to drought, soybean growth is restricted and more carbohydrates are stored [34]. Additionally, we found that sugar compounds such as ribose, deoxyribose, gluconate, xylose and xylitol are also increased during drought stress; however, the increase is relatively lower during heat stress, which indicates that heat stress will have a greater impact on amino acid metabolism and nucleic acid biosynthesis (Figure 3 and Figure S3, and Table S2). A comparatively recent study in soybean also indicated that under drought conditions, levels of the individual sugars varied considerably among the genotypes and among tissue types; the effect was found to be more pronounced in the leaves than in nodules [8]. Similarly, a transcriptomic profiling of soybean (cv. BR16) during water deficit is also in agreement with the finding of significantly increased transcripts for sugars during water deficit conditions [35].

### 2.3. Effect of Drought and Heat Stress on Amino Acid Metabolism

The metabolism of amino acids includes various biosynthetic reactions by which they are assembled as precursors of polypeptides and broken down to recuperate metabolic energy [36]. Under control conditions, during the daytime, photosynthesis provides the majority of the energy to the plants, and during the night, certain metabolites, primarily starch and amino acids, drive the plant’s energy generation processes via glycolysis, the TCA cycle, and amino acid metabolism [37,38]. 

**Figure 4:**
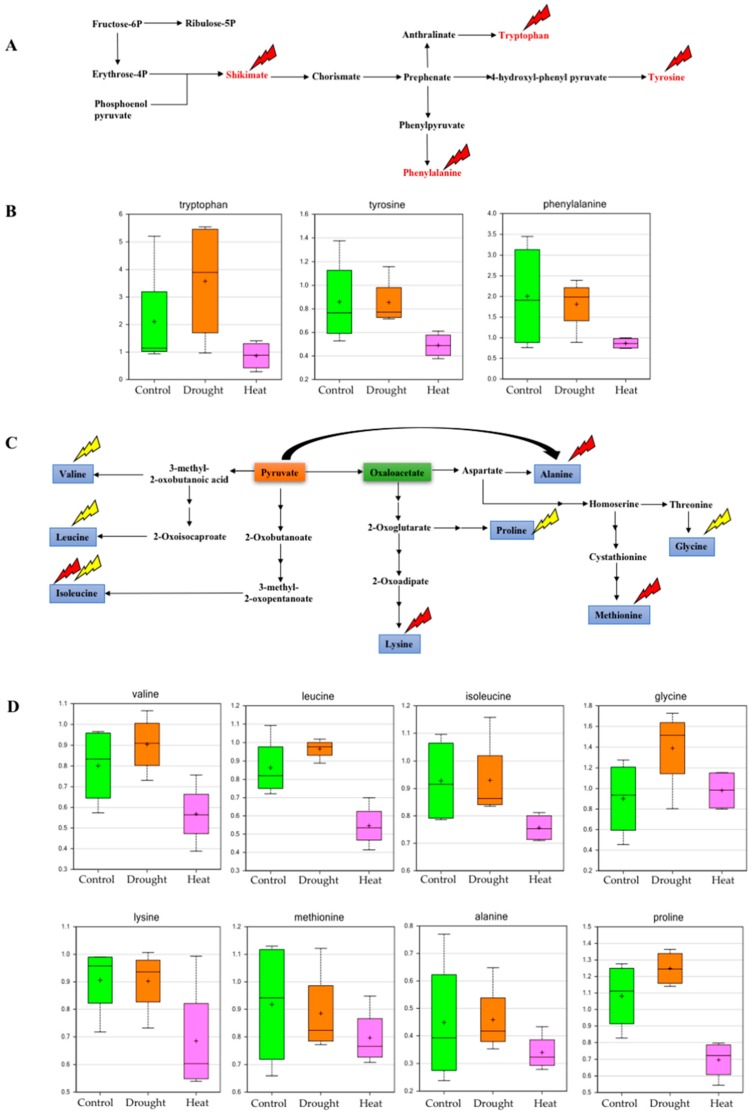
Effect of drought and heat stresses on metabolites of aromatic amino acid metabolism. (**A**) Pathway for aromatic amino acid metabolism showing the metabolites that were found to have significant effect of stresses on their abundance in the study (**B**) Box plots showing the relative abundances of aromatic amino acids in control, drought, and heat stress conditions (**C**) Effect of drought and heat stress on pyruvate and oxaloacetate-derived amino acid biosynthesis (**D**) Box plots showing relative levels of pyruvate and oxaloacetate-derived amino acids in control, drought and heat stress conditions. (Box plot description is the same as in Figure 2; red and yellow lightning signs in (**A**) and (**C**) indicate the effects of heat and drought stress, respectively).

Exposure of plants to drought and heat stresses reduces the photosynthesis rate, resulting in reduced growth and several physiological abnormalities. Convergent signal transduction associated with energy production regulates the response of the plants’ metabolome to various stresses [39]. Phosphoenolpyruvate (PEP) and erythrose-4-phosphate, which are main precursors of the aromatic amino acids, form 2-keto-3-deoxy-d-arabinoheptulosonate-7-phosphate by condensation reaction, eventually producing chorismate. Chorismate is the branch point for the synthesis of all aromatic amino acids (tryptophan, tyrosine and phenylalanine) [40]. The current study indicated that heat stress significantly affected aromatic amino acid metabolism. One of the precursor compounds of the aromatic amino acid biosynthetic pathway, shikimate, was greatly reduced during heat stress, which ultimately affected tryptophan, tyrosine and phenylalanine biosynthesis (Figure 4, and Table S2). The aspartic acid (Asp) family (oxaloacetic acid and pyruvate derived) of biosynthetic pathways leads to the biosynthesis of essential amino acids. i.e., lysine, alanine, threonine, methionine and isoleucine [37].

Metabolic regulation between the Asp-family pathway and the TCA cycle has a key impact on the physiological response of plants to drought and heat stresses. In our analysis, we also observed that all aspartate family-derived amino acids such as lysine, alanine, methionine and isoleucine, were reduced in abundance in response to heat stress (Figure 4, and Table S2). Recent studies also show that lysine is a precursor for a metabolic pathway that functions in plant development, and in response to various stresses [41].

It has been reported that alanine, one of the essential amino acids, is activated in response to flooding stress in *Lotus japonicus*, and is involved in regulating the TCA cycle, including anaplerotic and cataplerotic fluxes [42,43]. Increases in valine and leucine upon drought stress can also result from the consistent increase in the substrate pyruvate, which also featured in our GC-MS-based results [44]. The relative increase of isoleucine and leucine (Figure 4, and Table S2) in drought-stressed soybean leaves were in agreement with previous findings in Arabidopsis [45]. Glycine and proline are two major organic osmolytes that were highly accumulated (Figure 4) in response to drought stress [46,47]. Very high accumulation of cellular proline and glycine under drought stress was also observed in our study, which clearly shows the potential for an advanced tolerance response during adverse environmental conditions, such as drought, in soybeans. Thus, proline accumulation under drought stress might function as a sink for excess products that are necessary for the maintenance of photosynthetic and respiratory processes [47]. Similar findings specifically for amino acid metabolism, were presented by Tripathi et al. (2016) where metabolomic alterations during drought stress were monitored in hydroponics-grown soybeans [48].

### 2.4. Effect of Abiotic Stress on Nitrogen Metabolism

In agreement with proteomic findings (Das et al. [49]), a direct correlation for key amino acids such as glutamine and glutamate was reflected in this metabolomic investigation. The proteomics study [49] indicated that cytosolic glutamine synthetase, which assimilates ammonium to glutamine using glutamate as a substrate, is reduced significantly during drought, and alleviates nitric oxide (active radicals) levels to activate drought tolerance mechanisms in soybeans. Our metabolic data supports proteomic findings, as glutamate was found to be reduced during drought stress. By combining this metabolomics data with published proteomics data, we can make an inference that with lower levels of glutamine synthetase during drought stress it is difficult for soybean plants to assimilate ammonia, which in turn results in an abundance of glutamine (Figure S4). Interestingly, based on proteomics data [49] during heat stress, when glutamine synthetase is abundant, it helps the plant to use up the glutamate rapidly, in order to generate ample amounts of glutamine. 

### 2.5. Differential Response in Peptide Metabolism

Metabolites are the structural intermediates between amino acids and proteins during biological inter-conversions between these two groups of molecules [50]. It has been reported that severe oxidative stress induces protein mistranslation through the impairment of aminoacyl-tRNA biosynthesis [51]. One of the inevitable consequences of drought stress in plants is enhanced reactive oxygen species (ROS) generation and ROS-mediated degradation of the photosynthetic machinery during moderate heat stress [52,53]. Our analysis of amino acid metabolism suggested severe defects in the level of accumulation of cellular amino acids in response to drought and heat stress, which may directly reflect the rate of peptide synthesis via amino acyl tRNA biosynthesis.

**Figure 5:**
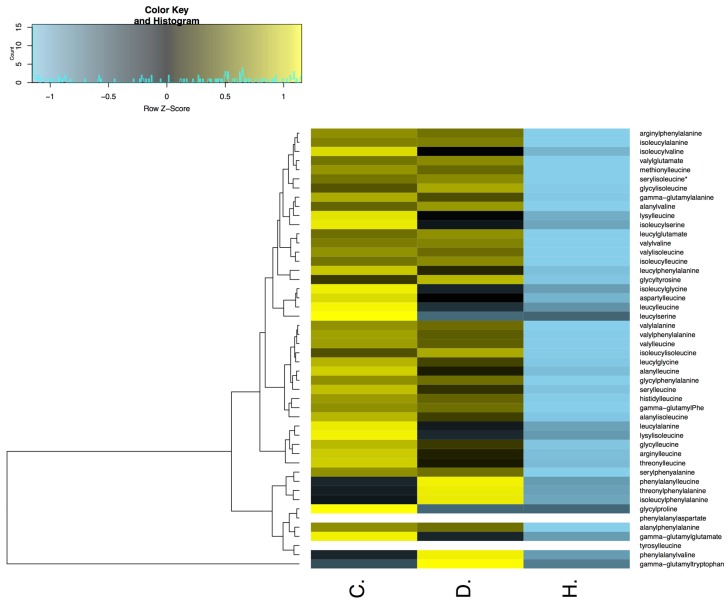
Differential abundance of peptides under drought and heat stresses. Heat map showing the differences in the abundance of the various peptides in soybean leaves under control (C.), drought (D.), and heat stress (H.) conditions.

We have found a relatively lower accumulation of major gamma-glutamyl peptides, namely gamma-glutamylalanine, gamma-glutamylglutamate, gamma-glutamylphenylalanine and gamma-glutamyltryptophan, upon drought and heat stress (Table S1). The results indicated a direct correlation between amino acid synthesis and dipeptide synthesis, especially during heat stress when we found that almost 81% of the detectable dipeptides were at a relatively low level compared to the controls (Table S1). Furthermore, during drought stress, only 47% of detectable dipeptides were lowered compared to controls (Table S1). Others were at a similar level or were higher than the control, which suggests that the plant protects against the adverse effects of drought stress by activating a tolerance mechanism to alleviate translation without any impairment of aminoacyl-tRNA biosynthesis. The heat map analysis shows the differential expression of all the identified peptides upon drought and heat stress (Figure 5).

### 2.6. Effect of Drought and Heat Stress on Nucleotide Metabolism and Protein Synthesis

Nucleotide metabolism is one of the essential components of plant metabolism and development, which affects many metabolic pathways [54]. 

**Figure 6:**
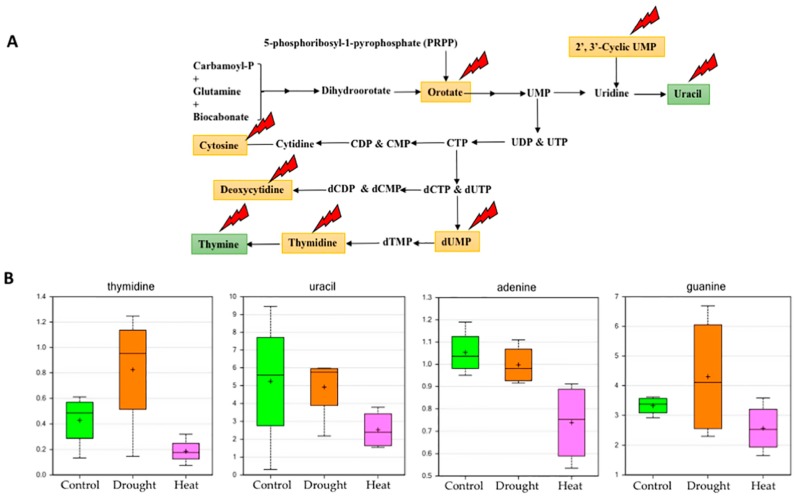
Effect of heat stress on regulation of pyrimidine metabolism. (**A**) Pathway for pyrimidine metabolism showing the metabolites that were found to have a significant effect of stresses on their abundance in the study. (**B**) Box plot showing major purine and pyrimidine bases and their differential abundances under control, drought and heat stress (Box plot description is the same as Figure 2; red lightning sign in (**A**) indicates heat stress).

Specifically, purine and pyrimidine nucleotides directly participate in nucleic acid synthesis, which provides the ultimate energy source for the synthesis of carbohydrates, lipids, peptides and secondary metabolites [55]. The de novo pyrimidine biosynthetic pathway (orotate pathway) is defined as the formation of uridine monophosphate (UMP)from carbamoyl phosphate (CP), orotate, 5-phosphoribosyl-1-pyrophosphate (PRPP), and a few other phosphate metabolites [56]. Our metabolomics profiling result indicates that the major building blocks, which are pyrimidine bases such as thymine, cytosine and uracil, are reduced significantly upon heat stress, which affects overall pyrimidine biosynthesis and nucleic acid biosynthesis (Figure 6).

Additionally, other precursor molecules of the pyrimidine biosynthetic pathway, such as thymidine, deoxycytidine, orotate, 2′,3′-cyclic UMP and dUMP are also reduced due to heat stress. Conversely, during heat stress, pyrimidine bases and precursor molecules are increased compared to the control, indicating the activation of a tolerance mechanism to protect nucleic acids and ultimately protein synthesis. The de novo purine biosynthetic pathway consists primarily of key amino acids, such as glutamine, aspartate, and glycine, activated ribose precursor PRPP, 10-formyl tetrahydrofolate, and CO_2_ [54]. The results from the current investigation show that upon heat stress, major purine bases such as adenine and guanine are downregulated compared to the control, along with small precursor molecules, such as 2′-deoxyadenosine, 2′-deoxyguanosine, and guanosine-2′,3′-cyclic monophosphate (Figure 6). Similarly, upon drought stress, the relative levels of all metabolites in purine biosynthesis increased, clearly indicating that the drought tolerance machinery is activated during stress conditions. The 2′,3′-cNMPs were more variable in soybeans, especially in the drought stressed samples. However, under heat stress, they appeared to respond in a similar manner to that observed for wheat, i.e., lower relative to the control. As for 3′,5′-cGMP, it was not detected in the drought stressed samples, and may have been lower in heat stress than in the control, although the variation in the control was high. 

### 2.7. Differential Regulation of Secondary Metabolites in Response to Drought and Heat Stress 

Studies on plant secondary metabolites indicate that they play a major role in the adaptation of plants to the changing environment and in overcoming various stress constraints [57,58]. It has been reported that heat stress greatly reduces the leaf area, which is an immediate response to heat. This effect is alleviated by the synthesis of vital amino acids and secondary metabolites [59]. Furthermore, studies specify that various polyphenols, particularly flavonoids and hydroxycinnamates accumulate at a higher level under drought-stress conditions to normalize the CO_2_ assimilation rate in the leaves [60].

**Figure 7:**
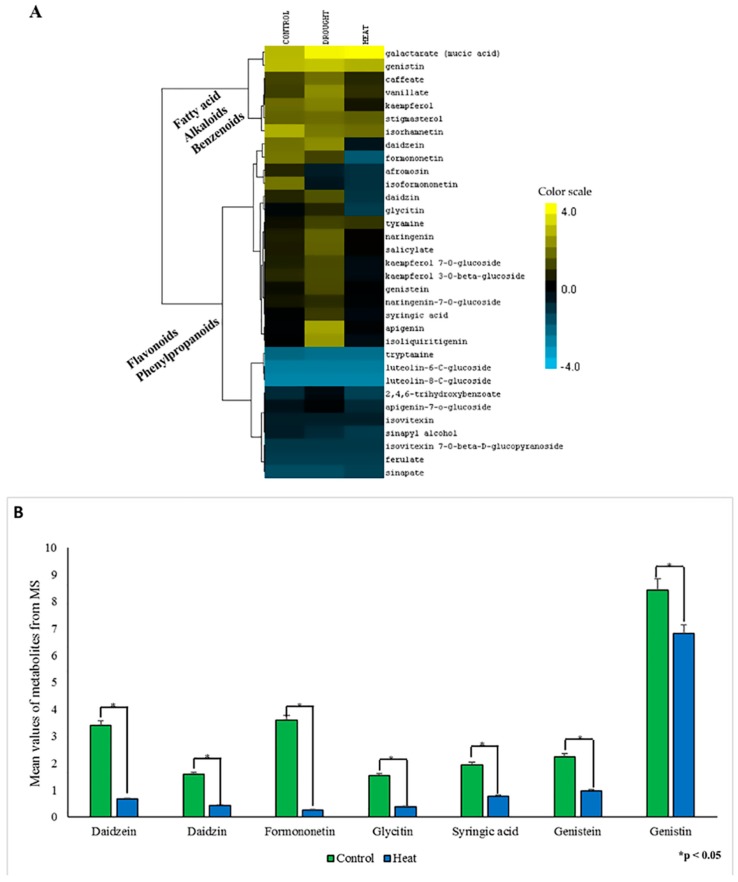
Effect of drought and heat stress on secondary metabolites. (**A**) Heat map showing differential expressions of various secondary metabolites during drought and heat stress compared to the control treatment. (**B**) Bar diagram showing relative expression of various phytochemicals in response to heat stress (blue) compared to the control (green). Various phytochemicals are shown on the *X*-axis, and the Y-axis has the scaled intensity of the respective phytochemical. Each value represents the mean ± S.E., and the asterisks designate the significance of changes from their corresponding control (*p* < 0.05).

In this metabolomic profiling, we found that the alkaloid caffeate accumulated at a comparatively higher level (1.5-fold) in response to drought stress than control plants, thus assuaging growth-related adversities and lignin synthesis [61]. One important benzenoid, vanillate, which is transformed to guaiacol and facilitates lignin biosynthesis, has a two-fold higher level during drought stress, but is at lower levels in heat stressed plants compared to the control plants. Flavonoid biosynthesis has been revealed to be upregulated by drought-induced oxidative stress, whereas photosynthesis is downregulated concomitantly [62]. This metabolic profiling also indicated that 73% of detected flavonoids and phenylpropanoids are upregulated upon drought stress, supporting the drought responsive role of flavonoids in soybeans (Figure 7A). Conversely, in response to heat stress, 84% of the detected flavonoids and phenylpropanoids accumulated at a lower level compared to the control, which indicates that during heat stress soybeans, especially cv. Williams-82, struggle to activate defense mechanisms effectively to relieve stress. 

### 2.8. Differential Expression of Soybean Phytochemicals

Soybeans are considered as one of the plant that produces a vast amount of isoflavones, which have profound effects as antioxidants [63]. Hosseinian et al. reported that in purple wheat, the anthocyanin content is increased due to heat stress, suggesting that abiotic stress may affect plant phytochemicals [64]. Conversely, studies with lettuce showed that they respond to heat stress by inducing the synthesis of various phytochemicals that act as antioxidants to activate defense mechanisms [65].

Interestingly, our results showed a significant reduction for some of the major soybean phytochemicals (daidzein, daidzin, formononetin, glycitin, syringic acid, genistein and genistin) in response to heat stress (Figure 7B). Thus, it can be implicated that heat stress affects the synthesis of these essential soy phytochemicals, and therefore diminishes the antioxidant capability of the plant. Conversely, our results showed that in response to drought stress, the same phytochemicals increased compared to the control (Figure S5), suggesting that plants might be activating their tolerance mechanisms and antioxidant activity to alleviate the stress [66]. The findings, especially the abundance of various phytochemicals (daidzein, daidzin, formononetin, syringic acid, genistein and genistin) in response to drought stress, are also supported by recent studies [48].

### 2.9. Metscape Analysis of Metabolomics Data Derived from LC- and GC-MS

For understanding of biological functions via usage of the computational approach, it is desirable to elucidate all functional metabolic interactions undergoing in the cell at a given time. There were many cellular compounds which were not detected in the metabolomics experimental pool for various reasons, for example, poor extraction or extremely low abundance, but they may have been be functionally associated with the detected compounds during drought and heat stress. MapMan and Metscape have the capability of illustrating this using a chart approach, to show an overall possible scenario for all of the related cellular compounds. Here we displayed a variety of functional metabolic interactions between the differentially expressed metabolites in soybeans in response to drought and heat stress, to obtain an improved understanding of metabolic pathways and metabolic regulation (Figure S6). Figure S7 has 27 input metabolites (red color) demonstrating how various carbohydrates are involved in glycolysis, TCA cycle, pentose phosphate pathway and other significant biological processes, and functionally interact with other related metabolites, and thus eventually may have an effect on biological and cellular processes when the soybean is exposed to stress. Figure S7 also shows related metabolites (yellow color), which could also be present in the same pathway as that of the input metabolites, such as pyruvate and oxaloacetic acid (OAA) of the TCA cycle. Thus, this network predicted that if a stress affected an input metabolite abundance, the abundance of corresponding related metabolites that were in the same pathway could also be affected as a result. Similarly, Figure S8 with 30 input amino acids shows related metabolites for amino acid biosynthesis metabolism. In sections above, we have discussed how drought and heat stress affects amino acid metabolism at various levels; in extension to that, this network predicts how the same pathway-derived undetected metabolites can also be affected by the same stress.

## 3. Materials and Methods 

### 3.1. Plant Growth Conditions 

Soybean (*Glycine max* L. cultivar: Williams-82 [67]) seeds were surface sterilized using 50% Clorox and planted in pots filled with Metro mix-360 (Sun Gro Horticulture Agawam, MA, USA). A total of nine pots (14 L volume; C1600, NurserySupplies.com) consisting of three groups (control, drought, and heat stress) with three replications of each in complete randomization were planted in a growth chamber (Conviron, Winnipeg, MB, Canada) illuminated with white fluorescent and incandescent bulbs (500 μmoles m^−2^ s^−1^, 16 h of light), at 28/24 °C and 60% relative humidity. Each pot had four individual soybean plants, and the stress treatments were performed as explained in Das et al. [49]. Briefly, watering was done daily, and as needed, with tap water until the second trifoliate leaves emerged. At this stage, the nine pots were separated into three groups where each group contained three treatments as follows: control, drought, and heat stress. In the first ‘control’ group, plants were maintained under water-saturated soil conditions at 28/24 °C. In the second ‘drought’ group, the plants were not watered, and gradually reached 10% volumetric water content (VWC) on the seventh day of the experiment under 28/24 °C. A separate growth chamber contained pots that were exposed to high temperatures (43/35 °C with 50% humidity) with water saturated soils; these were the third ‘heat’ group. After the seventh day of treatments, young trifoliate leaves from developmentally matched plants (number of plants >3) were harvested at noon in bulk from one pot as one replication unit, based on previously published studies in Soybean [49], and were immediately snap-frozen in liquid nitrogen and stored at −80 °C. 

### 3.2. Metabolomic Profiling 

Metabolomic profiles were analyzed using harvested soybean leaves. Leaf samples ground in liquid nitrogen were submitted to Metabolon, Inc. (Durham, NC, USA). The sample preparation and analysis process was performed as previously described [68,69,70]. Briefly, samples were extracted in 400 μL of methanol using an automated liquid handling system; the resultant samples were split into three aliquots for analysis on three MS instrument platforms. These included two UPLC/MS platforms, one optimized for positive ionization and a second optimized for negative ionization. The third aliquot was derivatized and analyzed by GC/MS. The UPLC-MS/MS platform included a Waters ACQUITY ultra-performance liquid chromatography (UPLC) system, a Thermo Scientific Q-Exactive high resolution/accurate mass spectrometer with heated electrospray ionization (HESI-II) source, and an Orbitrap mass analyzer operated at 35,000 mass resolution. The MS scan range was from 80 to 1000 *m*/*z*. GC samples were analyzed on a Thermo-Finnigan Trace DSQ fast-scanning single-quadrupole mass spectrometer using electron impact ionization (EI). The MS scan range was from 50 to 750 *m*/*z*. The mass spectrometry peaks were identified using Metabolon’s proprietary peak integration software. Peaks were quantified using the area-under-the-curve method. The identification of known chemical entities was based on comparisons to metabolomic library entries of purified standards [71]. Because the analysis spanned multiple days, a data normalization step was performed to correct any variation resulting from instrument inter-day tuning differences. Essentially, each compound was corrected in run-day blocks by setting the medians equal to one (1.00) and normalizing each data point proportionately. Since all the treatments were comprised of three replicates (n = 3 for all groups), the statistical significance of the results was evaluated using Welch’s two sample t-test and a level of significance of *p* ≤ 0.05 for two group comparisons.

### 3.3. Data Analyses 

The log2-transformed values of the metabolites mean abundances were used to cluster the metabolomic data. The heat maps and hierarchical clustering of the metabolites were performed using Gene Cluster 3.0 software [72]. The metabolite clusters were visualized using JAVA TREEVIEW software [73]. The statistical significance of the results was evaluated using Welch’s two sample *t*-test. A value of *p ≤* 0.05 was considered statistically significant for two-group comparisons. To visualize the relationships of metabolites expression levels in control versus stress (drought or heat) a scatter plot was constructed using R 3.3.1 software [74]. MapMan application software (MapMan Version 3.5.1R2) was used for understanding the metabolic distributions and metabolic regulation in response to the stresses [75]. Selected metabolomic interactions were portrayed using MetScape 3.1 plugin for Cytoscape; we also displayed several protein networks using Cytoscape software, Version 3.0.1, and the KEGG database was used for ID referencing [76,77,78].

## 4. Conclusions

Metabolomic analysis of soybean leaves revealed a dynamic alteration in metabolites in response to drought and heat stress. This analysis clearly demonstrates the changes in metabolic pathways that may affect soybean growth and development. However, in response to heat stress, the soybean plants were severely impacted. From a metabolomic standpoint, we found that several metabolites were downregulated during heat stress, complicating the homeostasis of the general metabolism of the soybean plant. A detailed combined proteomic-transcriptomic study using soybean roots, showed that proteins involved in amino acids, and lipid biosynthesis were severely affected under heat stress [79]. This study corroborates our metabolomics findings in response to heat stress. However, an opposite scenario was observed during drought stress, where most of the metabolites significantly increased in abundance or remained the same as the control plants in our investigation. Interestingly enough, a similar reflection was observed during a previous transcriptome-based study on soybean (cv. BR16, and cv. Williams 82), which revealed that several genes, involved in nucleotide metabolism, amino acid biosynthesis, lipid metabolism secondary metabolism, were significantly increased during drought stress compared to the control [35,80]. Another study using drought-tolerant (cv. Embrapa 48) and drought-sensitive (cv. BR16) soybeans indicated that under drought stress, more than 50% genes related to various metabolic processes increased in expression independent of the tolerance factor [81].

**Figure 8:**
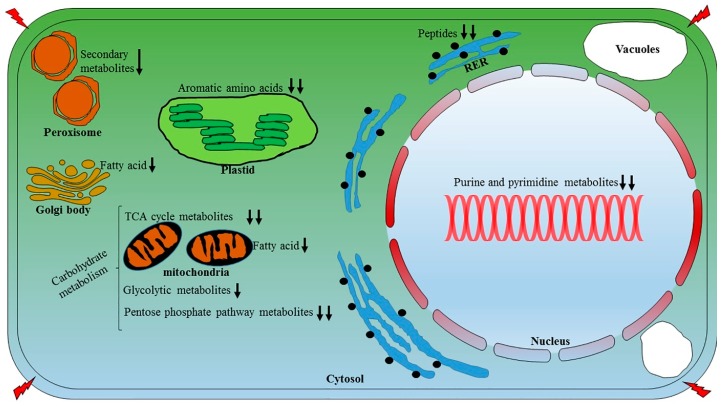
A proposed model shows the metabolomic responses of soybean leaves during heat stress. The figure shows the effects of heat stress on key metabolites in key cell organelles. Single and double downward-pointing arrows (based on scaled quantity of metabolites) indicate metabolites reduced due to heat stress. There was no metabolite found in this investigation that showed increased abundance during heat stress. The red lightning arrows on the corners of the cell represent heat stress.

Based on metabolomic profiling and using a MapMan-derived metabolic pathway overview (Figure S6), we proposed a model (Figure 8), which explains the heat stress-related changes in soybeans. The present study showed that in response to drought stress, soybeans experience reduced growths to alleviate the stress effects. In addition, we suggested that during drought, soybeans try to activate a tolerance pathways to ensure an adjusted metabolism, which is likely one of the soybeans defense strategies, similar to other plants, for combating drought stress [82,83]. By using compounds that were detected by GC- and LC-MS as input data, we also built regulatory networks, addressing related compounds that were not detected but might be affected in response to abiotic stress (Figures S6 and S7). Likewise, we also found that heat stress affects the synthesis of phytochemicals, such as daidzein, daidzin, formononetin, glycitin, syringic acid, genistein and, genistin.

In addition to revealing the dynamic alteration in metabolic pathways, metabolomic approaches also provide a parallel insight into metabolite composition that can be used for crop improvement projects [22]. Recent studies in tomatoes using comprehensive metabolic profiling uncovered traits for improved crop quality [84]. Metabolite profiling using NMR and GC-MS studies also revealed potentially unintended effects in genetically modified tomatoes [85]. Studies have also reported the emerging importance of using metabolites as selection markers for crop breeding, as metabolite biomarkers are associated with relevant traits under environmental stresses [12,86,87,88]. 

This study provides information on several metabolites that include major carbohydrates, amino acids, nucleotides and soybean phytochemicals, which can be useful for the development of better models to establish the connection between yield-associated traits and various metabolic pathways. The present study also has potential to support metabolomics-assisted breeding for the development of more stress-tolerant soybean varieties. The report, combined with high-throughput reverse genetics studies, can also assist in the rapid development of elite soybean lines. This study identifies a detailed mechanism of metabolic alteration in soybeans in response to drought and heat stresses.

## Supplementary Materials

The following are available online at www.mdpi.com/2223-7747/6/2/21/s1, Figure S1: Categorization of identified metabolites in seven broad groups. *Y* axis has number of metabolites; *X* axis has 7 different broad categories of identified metabolites. Green color bar represents number of metabolites that were found increased in abundance; golden color bar represents number of metabolites decreased in abundance compared to the control condition, Figure S2: Effect of stress on metabolites involved in starch and carbohydrate metabolism (A) Box plots showing the effect of drought and heat stress on the expression of maltose, galactitol, sucrose, and mannitol. (B) Heat map showing the effect of drought and heat stress on the abundance of metabolites that are involved in starch and carbohydrate metabolism, Figure S3: Metscape network shows the effect of abiotic stress on sugar metabolism. Red colored nodes indicate input metabolites from GC/LC-MS results, green squares indicate enzymes, grey squares indicate pathway number from KEGG database and other hexagons indicate other predicted metabolites that might be associated with the reaction, Figure S4: A proposed model by combining proteomic and metabolomic studies in the realm of nitrogen metabolism. Figure shows how glutamate and glutamine metabolite level is regulated in response to drought and heat stress based on differential expression of glutamine synthetase. (Based on Das et al., 2016 [49], and this study), Figure S5: Differential expression of phytochemicals in response to drought stress, Figure S6: Metabolic pathways regulation in response to heat stress derived from MapMan. 93 data points were mapped and 72 data points are visible in this figure. Arabidopsis ATH1_TAIR9 was used as mapping template, Figure S7: A fully connected network of all carbohydrate derived metabolites detected in response to heat stress. It is a compound-gene based network. Red color nodes indicate input data and yellow color nodes indicate related compounds with input compounds. This network comprises of 110 nodes and 217 edges, Figure S8: Network elucidating all amino acid derived metabolites detected in response to drought and heat stress. It is a compound-gene based network. Red color nodes indicate input data and yellow color nodes indicate related compounds with input compounds. This network comprises of 244 nodes and 502 edges, Table S1: Ratio for differential expression, fold change and mean values of metabolites in response to drought and heat stress compared to the control, Table S2: Mean and *p*-values of differentially expressed carbohydrates and amino acids. (A) Summary of differentially expressed carbohydrates during drought and heat stress compared to the control, along with its mean and *p*-values. A value of *p* ≤ 0.05 was considered statistically significant (red font) for the two group comparisons. (B) Summary of differentially expressed amino acids during drought and heat stress compared to the control, along with its mean and *p*-values. A value of *p* ≤ 0.05 was considered statistically significant (red font) for the two group comparisons.
