# Supplementary material for: Metabolomic Profiling of Soybeans (Glycine max L.) Reveals the Importance of Sugar and Nitrogen Metabolism under Drought and Heat Stress
Source: Plants (Basel). 2017 May 25;6(2):21. doi: 10.3390/plants6020021 (PMC5489793; doi:10.3390/plants6020021)
Supplement: Supplementary file 1 [file plants-06-00021-s001.zip › Table S2.docx]

**Supplementary Table 2A**: Summary of differentially expressed carbohydrates during drought and heat stress compared to the control, along with its mean and p-values. A value of p ≤ 0.05 was considered statistically significant (red font) for the two group comparisons.

| **Carbohydrates** | | **Mean value (µ)** | | | ***p-value*** | |
| --- | --- | --- | --- | --- | --- | --- |
| **Sub Pathway** | **Biochemical Name** | **Control**  **(C)** | **Drought**  **(D)** | **Heat (H)** | **t test**  **(C, D)** | **t test**  **(C, H)** |
|  | 1,3-dihydroxyacetone | 1.0826 | 1.7015 | 0.8941 | 0.0033 | 0.3096 |
| **Glycolysis** | Glucose | 0.6145 | 0.6625 | 0.5688 | 0.6154 | 0.0045 |
|  | Pyruvate | 3.6594 | 3.338 | 1.3684 | 0.05758 | 3.46e-^07^ |
| **TCA cycle** | Alpha-ketoglutarate | 5.2305 | 4.3049 | 2.5151 | 0.8797 | 0.0337 |
|  | Citrate | 3.8960 | 3.7769 | 3.0743 | 0.1112 | 0.0103 |
|  | Fumarate | 32.6296 | 45.8797 | 23.6894 | 0.0928 | 0.0655 |
|  | Malate | 1.7807 | 1.8116 | 1.3131 | 0.9793 | 0.0005 |
|  | Oxaloacetate | 0.3052 | 0.3664 | 0.0997 | 0.0184 | 3.30e^-05^ |
|  | Succinate | 4.4009 | 3.9595 | 2.7150 | 0.0187 | 3.81e^-05^ |
| **PP pathway** | 2-deoxyribose | 0.3407 | 0.6119 | 0.2446 | 6.91E-07 | 0.0016 |
|  | Gluconate | 1.1207 | 1.1327 | 0.5997 | 1.31E-06 | 1.43e^-05^ |
|  | Ribose | 1.3687 | 1.2855 | 1.1761 | 0.2689 | 0.0095 |
|  | Xylitol | 1.0872 | 1.3228 | 0.3149 | 0.2957 | 8.79e^-07^ |
|  | Xylose | 0.7133 | 0.8759 | 0.6106 | 0.4931 | 0.0251 |
|  | Glycerate | 1.1194 | 1.0077 | 1.4849 | 0.0070 | 3.64e^-06^ |

**Supplementary Table 2B**: Summary of differentially expressed amino acids during drought and heat stress compared to the control, along with its mean and p-values. A value of p ≤ 0.05 was considered statistically significant (red font) for the two group comparisons.

| **Amino acids** | | **Mean value (µ)** | | | ***p-value*** | |
| --- | --- | --- | --- | --- | --- | --- |
| **Sub Pathway** | **Biochemical Name** | **Control**  **(C)** | **Drought**  **(D)** | **Heat (H)** | **t test**  **(C, D)** | **t test**  **(C, H)** |
| **Aromatic** | phenylalanine | 2.0059 | 1.8117 | 0.8640 | 0.0056 | 7.12E^-06^ |
|  | tryptophan | 2.1063 | 3.5751 | 0.8655 | 0.0326 | 6.61E^-06^ |
|  | tyrosine | 0.8591 | 0.8542 | 0.4908 | 0.0191 | 0.0004 |
| **Aspartate family** | alanine | 0.4487 | 0.4588 | 0.3394 | 0.0162 | 0.0011 |
|  | lysine | 0.9059 | 0.9026 | 0.6850 | 0.2312 | 4.84E^-05^ |
|  | methionine | 0.9180 | 0.8851 | 0.7965 | 0.1667 | 0.0081 |
| **Branched Chain** | isoleucine | 0.9282 | 0.9300 | 0.7573 | 0.1013 | 0.0001 |
|  | leucine | 0.8633 | 0.9652 | 0.5461 | 0.0060 | 3.20E^-07^ |
|  | valine | 0.8013 | 0.9038 | 0.5681 | 0.0076 | 0.0005 |
| **Glutamate family** | Glutamate | 0.4491 | 0.356 | 0.5051 | 0.4038 | 0.0029 |
|  | Glutamine | 0.6518 | 0.7472 | 0.776 | 0.0289 | 0.0304 |
| **Others** | proline | 1.0813 | 1.2487 | 0.6964 | 0.0210 | 2.78E^-05^ |
|  | glycine | 0.9005 | 1.3897 | 0.9808 | 0.0001 | 0.3373 |
