# Supplementary figures and images for: Metabolomic Profiling of Soybeans (Glycine max L.) Reveals the Importance of Sugar and Nitrogen Metabolism under Drought and Heat Stress
Source: Plants (Basel). 2017 May 25;6(2):21. doi: 10.3390/plants6020021 (PMC5489793; doi:10.3390/plants6020021)

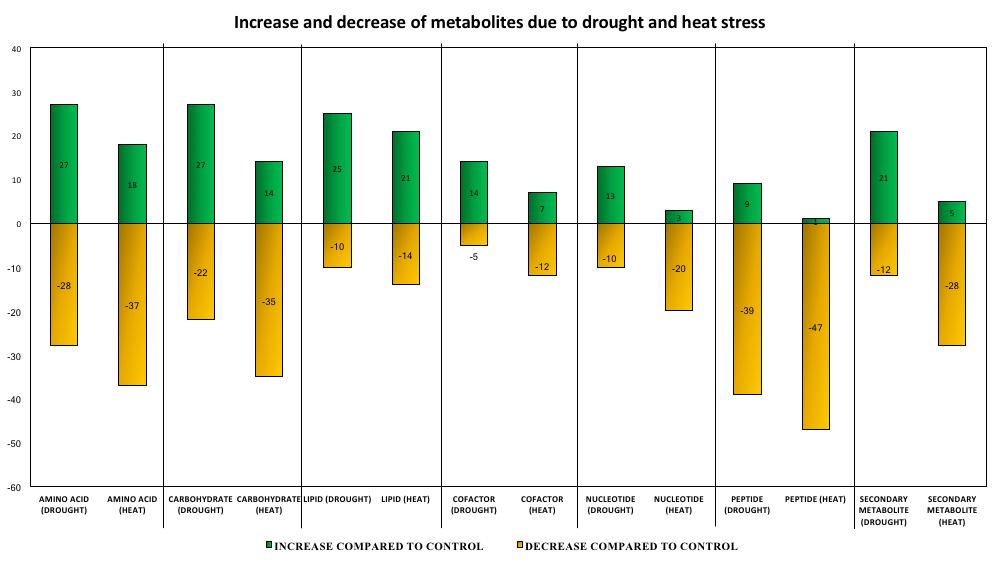

Supplement: Supplementary file 1 [file plants-06-00021-s001.zip › Supplemental figures TIF/Figure S1.tiff]

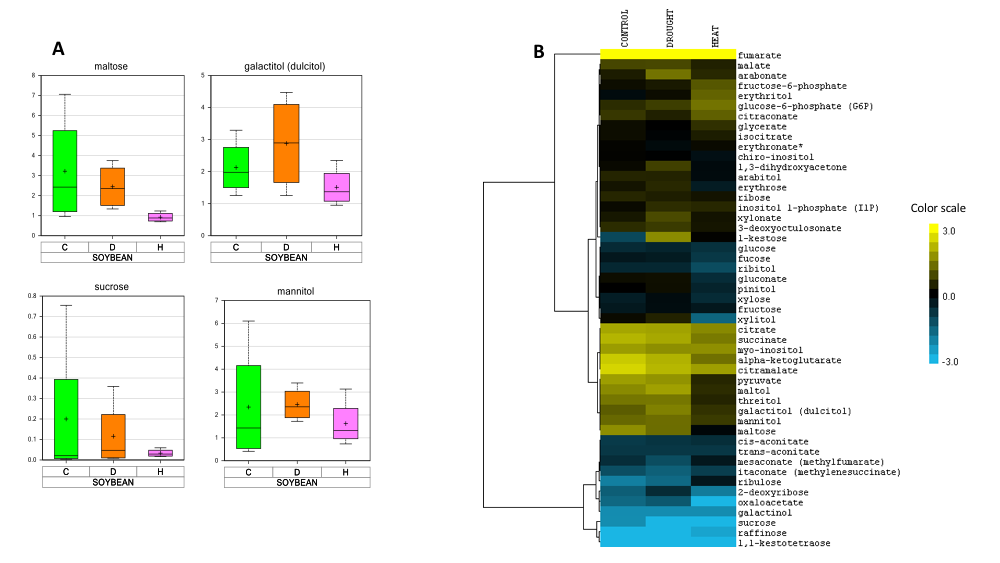

Supplement: Supplementary file 1 [file plants-06-00021-s001.zip › Supplemental figures TIF/Figure S2.tiff]

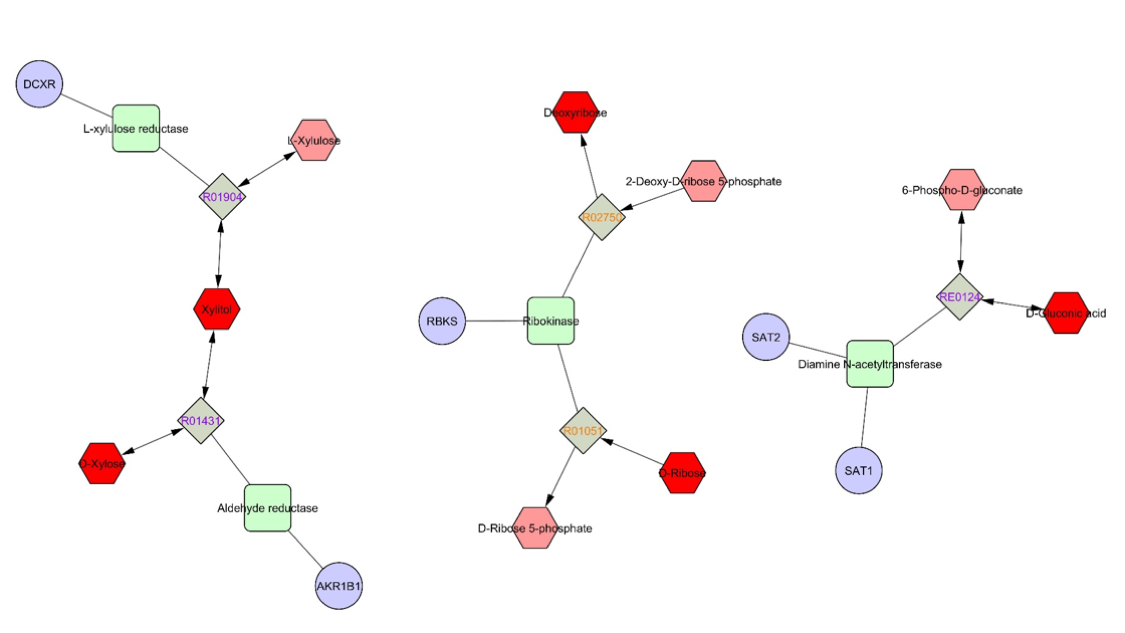

Supplement: Supplementary file 1 [file plants-06-00021-s001.zip › Supplemental figures TIF/Figure S3.tiff]

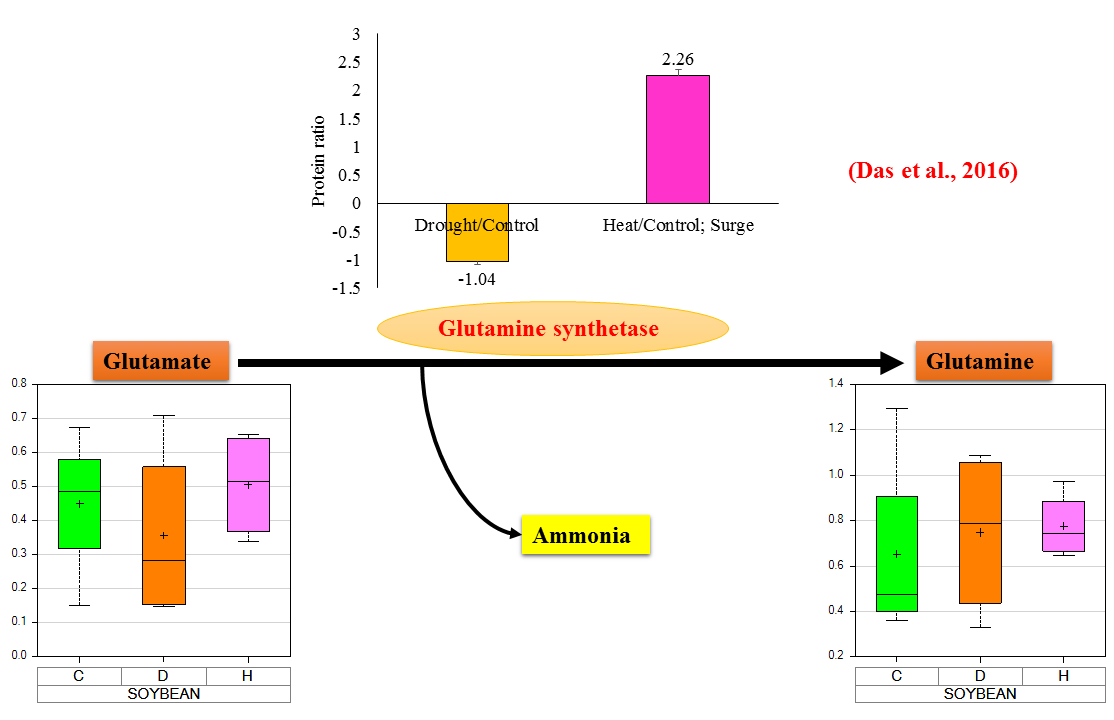

Supplement: Supplementary file 1 [file plants-06-00021-s001.zip › Supplemental figures TIF/Figure S4.tif]

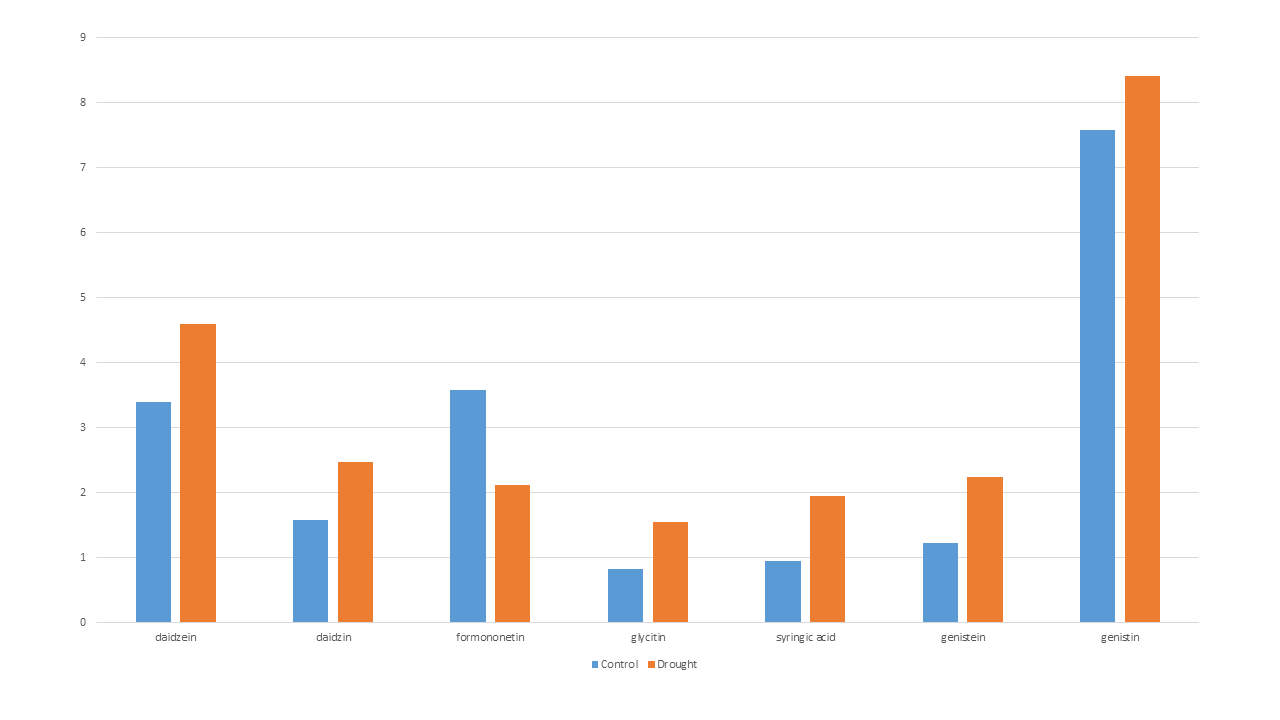

Supplement: Supplementary file 1 [file plants-06-00021-s001.zip › Supplemental figures TIF/Figure S5.TIF]

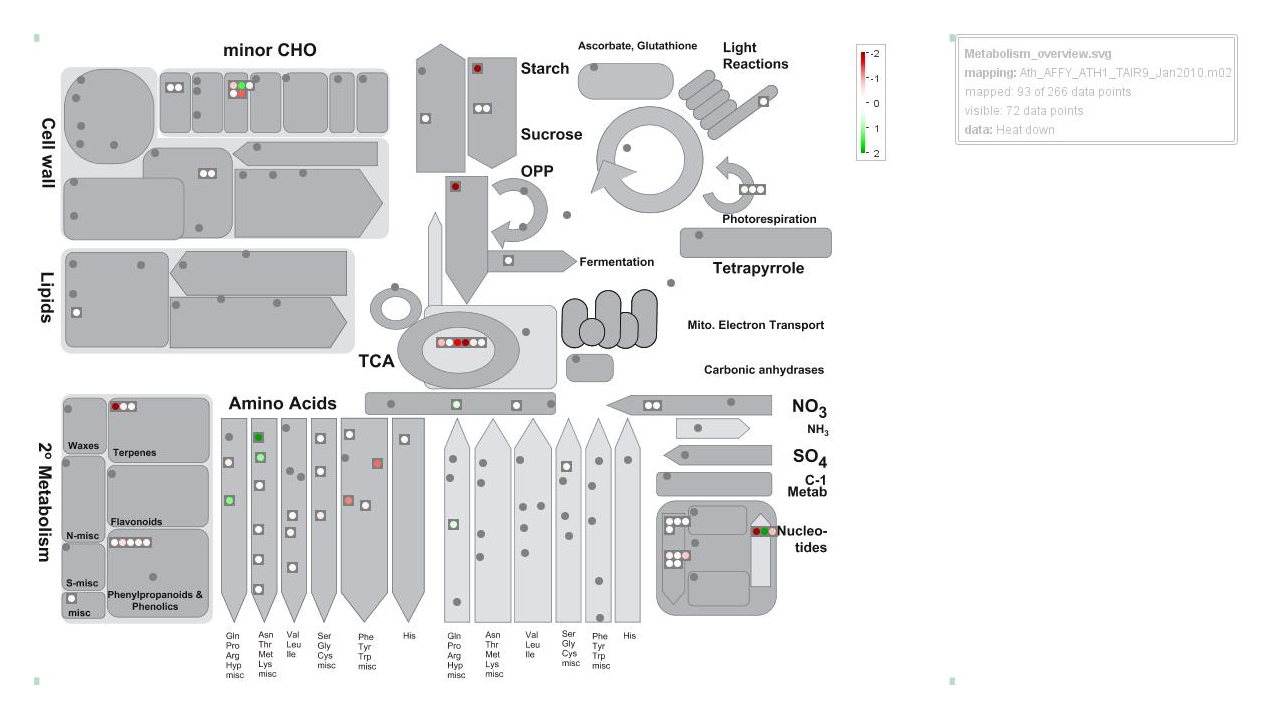

Supplement: Supplementary file 1 [file plants-06-00021-s001.zip › Supplemental figures TIF/Figure S6.tif]

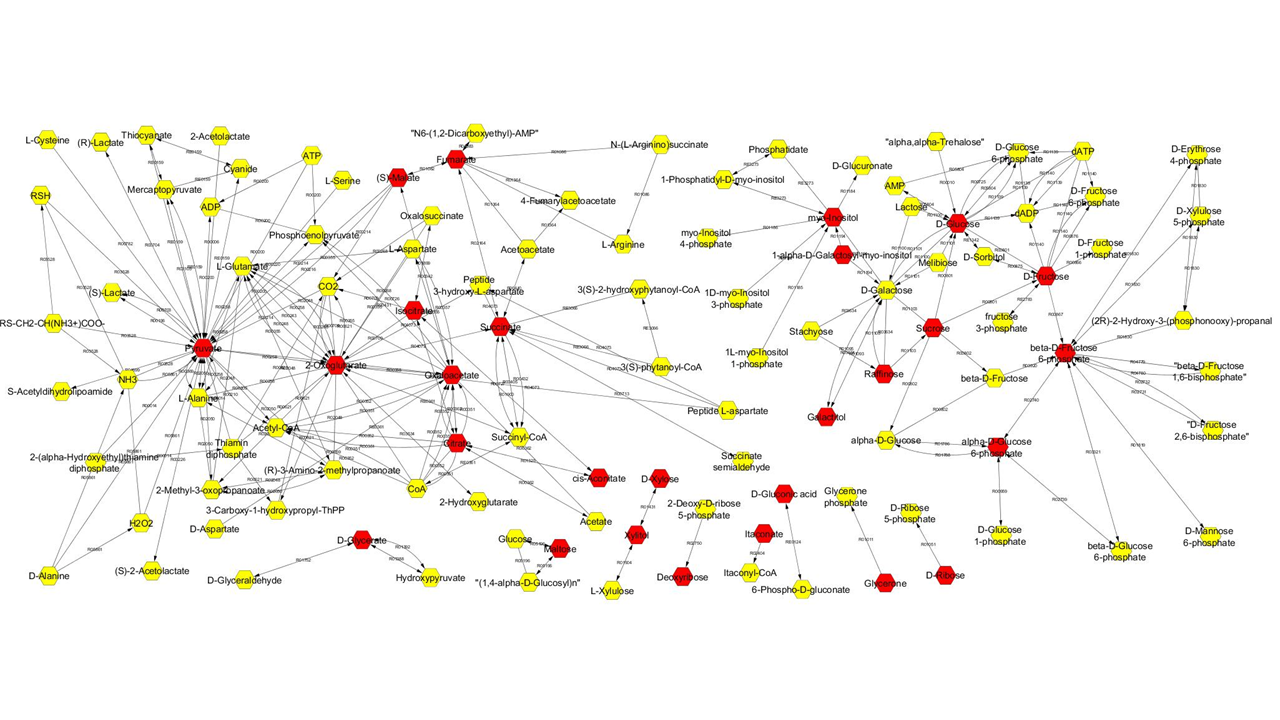

Supplement: Supplementary file 1 [file plants-06-00021-s001.zip › Supplemental figures TIF/Figure S7.tif]

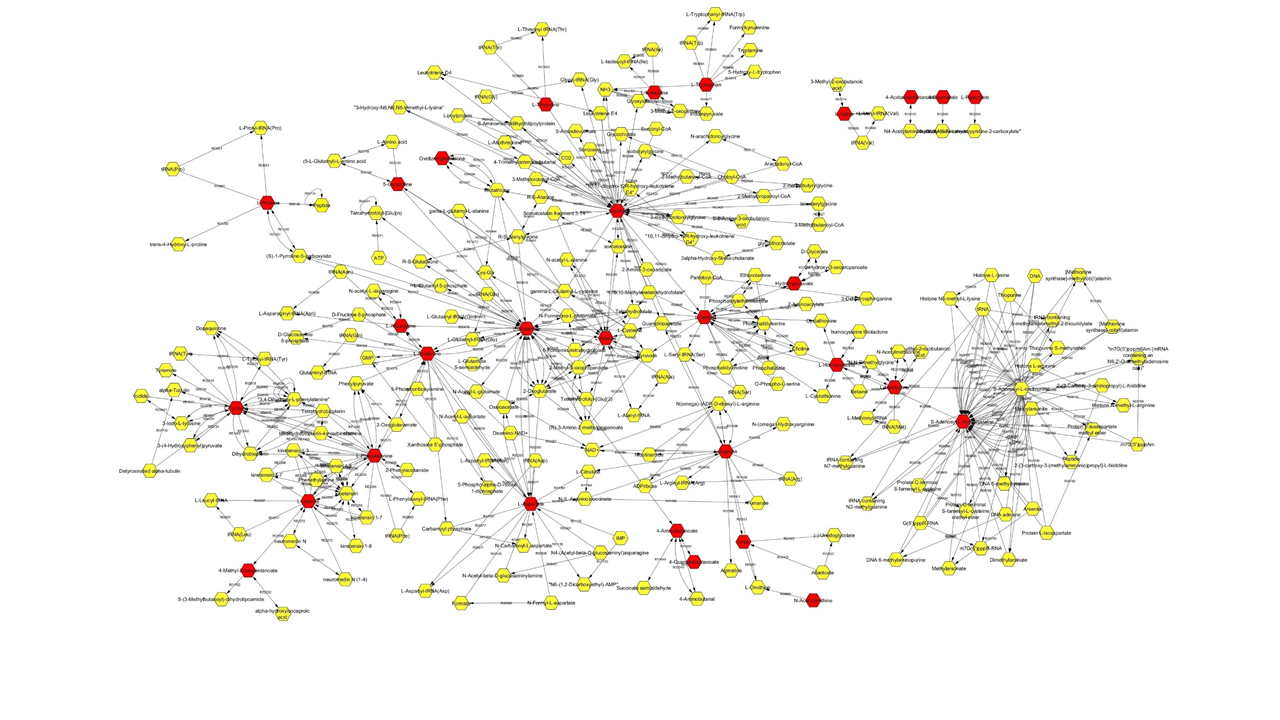

Supplement: Supplementary file 1 [file plants-06-00021-s001.zip › Supplemental figures TIF/Figure S8.tif]
